# Dr. William K. Otis

**Published:** 1906-11

**Authors:** 


Dr. William K. Otis, of New York, a graduate of the College of Physicians and Surgeons in 1885, died at his home after a short illness of pneumonia, September 22, 1906, aged 36 years. He was a son of Dr. Fessenden N. Otis and was following the general line of practice pursued by his distinguished father, and in which he himself was achieving fame. He was a member of many local and other societies and was professor of genitourinary diseases at the New York School of Clinical Medicine. He also had service in several hospitals.

